# NPC1L1 inhibitor ezetimibe is a reliable therapeutic agent for non-obese patients with nonalcoholic fatty liver disease

**DOI:** 10.1186/1476-511X-9-29

**Published:** 2010-03-12

**Authors:** Munechika Enjoji, Kazuyuki Machida, Motoyuki Kohjima, Masaki Kato, Kazuhiro Kotoh, Kazuhisa Matsunaga, Manabu Nakashima, Makoto Nakamuta

**Affiliations:** 1 Faculty of Pharmaceutical Sciences, Fukuoka University, 8-19-1 Nanakuma, Jonan-ku, Fukuoka 814-0180, Japan; 2 Clinical Research Center, Kyushu Medical Center, National Hospital Organization, 1-8-1 Jigyohama, Chuou-ku, Fukuoka 810-8563, Japan; 3 Department of Gastroenterology, Kyushu Medical Center, National Hospital Organization, 1-8-1 Jigyohama, Chuou-ku, Fukuoka 810-8563, Japan; 4 Department of Medicine and Bioregulatory Science, Graduate School of Medical Sciences, Kyushu University, 3-1-1 Maidashi, Higashi-ku, Fukuoka 812-5282, Japan

## Abstract

**Background:**

We recently examined the distribution of abdominal fat, dietary intake and biochemical data in patients with nonalcoholic fatty liver disease (NAFLD) and found that non-obese NAFLD patients did not necessarily exhibit insulin resistance and/or dysregulated secretion of adipocytokines. However, dietary cholesterol intake was superabundant in non-obese patients compared with obese patients, although total energy and carbohydrate intake was not excessive. Therefore, excess cholesterol intake appears to be one of the main factors associated with NAFLD development and liver injury.

**Methods:**

We reviewed a year of follow-up data of non-obese NAFLD patients treated with Niemann-Pick C1 like 1 inhibitor ezetimibe to evaluate its therapeutic effect on clinical parameters related to NAFLD. Without any dietary or exercise modification, 10 mg/day of ezetimibe was given to 8 patients. In 4 of 8 patients, ezetimibe was administered initially. In the remaining 4 patients, medication was switched from ursodeoxycholic acid to ezetimibe.

**Results:**

In each patient, body mass index was maintained under 25 kg/m^2 ^during the observation period. Serum ALT levels significantly decreased within 6 months and in 4 patients levels reached the normal range (<30 U/L), which was accompanied with at least a 10% decrease in serum total cholesterol and LDL-cholesterol. However, ultrasonographic findings of fatty liver did not show obvious improvement for a year.

**Conclusion:**

We conclude that the cholesterol absorption inhibitor ezetimibe can suppress hepatic injury in non-obese patients with NAFLD and that ezetimibe may offer a novel treatment for NAFLD.

## Background

Nonalcoholic fatty liver disease (NAFLD), which is characterized by hepatic steatosis, is a common cause of abnormal liver function and its incidence is increasing in many countries. Many NAFLD patients progress to a severe form of nonalcoholic steatohepatitis that can lead to cirrhosis, hepatic failure and hepatocellular carcinoma [1,2]. Although obesity and/or insulin resistance are considered to be a common cause of NAFLD, a large proportion of NAFLD patients are non-obese individuals [3,4]. Because onset and progression of NAFLD seem to be affected by nutritional intake, we have compared the distribution of abdominal fat, dietary intake and biochemical data between obese and non-obese patients with NAFLD to identify potential nutritional factors that affect NAFLD [5,6]. Waist circumference, total abdominal fat levels and subcutaneous fat levels were significantly higher in the obese group, whereas visceral fat levels were not significantly different between the two groups. Non-obese patients did not show overt insulin resistance and serum levels of adipocytokines were not abnormal. Although total energy and carbohydrate intake tended to be higher in the obese group, dietary cholesterol intake was significantly higher and intake of polyunsaturated fatty acids (PUFAs) was significantly lower in the non-obese group. Considering these results, suppressing cholesterol absorption may offer a novel strategy for the treatment of NAFLD patients. Accordingly, the Niemann-Pick C1 like 1 (NPC1L1) inhibitor ezetimibe was a suitable candidate to test this hypothesis. In this study, we evaluate the therapeutic effect of ezetimibe on non-obese NAFLD patients from the viewpoints of hepatic injury, dyslipidemia, and ultrasonographic fatty change. Ezetimibe had a prompt and excellent clinical effect on laboratory findings except for imaging.

## Patients and methods

**Figure 1:**
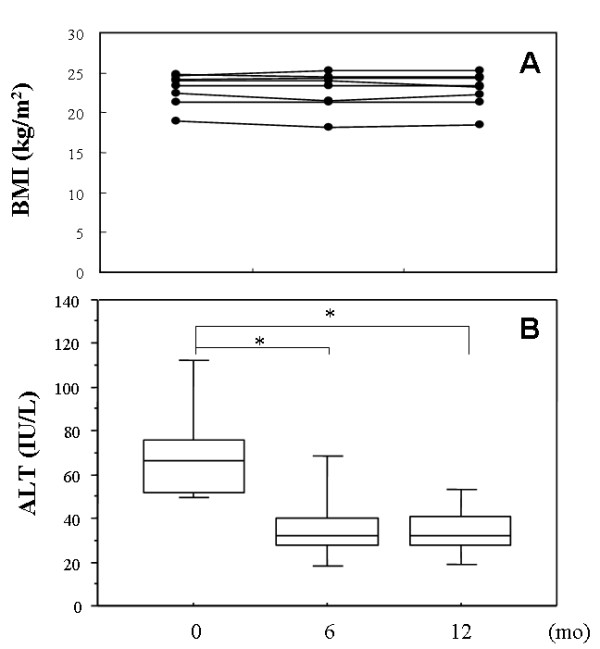
**Changes of body mass index (BMI) (A) and serum alanine aminotransferase (ALT) levels (B) during ezetimibe treatment**. Values at baseline, 6 months and 12 months are presented. *p < 0.05.

The study population included 8 patients (males:females = 6:2, age: 49.50 ± 10.76 years) with NAFLD who were diagnosed at Kyushu Medical Center Hospital between October 2007 and June 2008. All patients provided written informed consent before entering the study. They met the following criteria of NAFLD: (i) alcohol intake <20 g/day; (ii) exclusion of other liver diseases; (iii) the bright liver pattern with liver-kidney contrast and vascular blurring by echotexture, or the liver-to-spleen attenuation ratio <0.9 on computed tomography. Moreover, they were classified as non-obese based on a body mass index (BMI) of less than 25 kg/m^2^. In the evaluation of homeostasis model assessment-insulin resistance (HOMA-IR), no patients were classified as insulin resistance defined by HOMA-IR>2.5. Before enrollment, other etiologies of chronic liver disease were ruled out again in each patient. Neither dietary nor exercise therapy was prescribed, and their lifestyle was unchanged. Therefore, their body weight and BMI were kept stable during the following therapy (Figure 1A). Four of 8 patients had not been treated with any medicines for NAFLD and dyslipidemia, such as ursodeoxycholic acid (UDCA), fibrates or statins. Another 4 patients had received 600 mg/day of UDCA before ezetimibe treatment. For these patients, medication of ezetimibe (10 mg/day, orally) was started initially or to replace UDCA. No other agents for liver diseases, dyslipidemia or diabetes were used on the patients during ezetimibe treatment. Only one patient was hypertensive and the Ca-blocker, amlodipine, was continued. All of these patients have continued follow-up at our center with weight monitoring and estimates of physical activity. Statistical analysis was performed using the Wilcoxon signed rank-sum test (p < 0.05) and Pearson and Spearman correlations to compare biochemical variables. This follow-up study was approved by the Kyushu Medical Center Institutional Review Board for Human Subjects Research.

## Results

**Figure 2:**
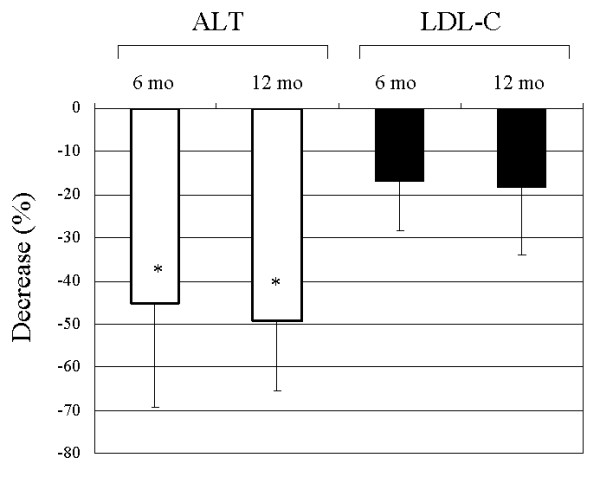
**Changes of serum alanine aminotransferase (ALT) and LDL-cholesterol (LDL-C) levels during ezetimibe treatment**. Decrease (%) from baseline is presented at 6 and 12 months after the treatment initiation. *p < 0.05 compared with baseline.

**Figure 3:**
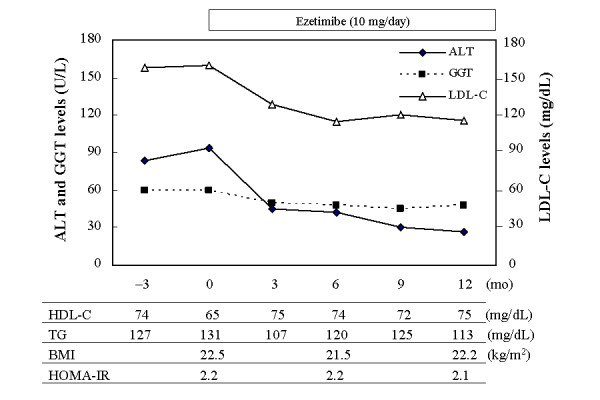
**Clinical course of a representative case**. ALT, alanine aminotransferase; GGT, γ-glutamyl transpeptidase; LDL-C, LDL-cholesterol; HDL-C, HDL-cholesterol; TG, triglyceride; BMI, body mass index, HOMA-IR, homeostasis model assessment-insulin resistance.

Background of the non-obese NAFLD patients examined is shown in Table 1. UDCA had been administered in 4 patients; however, it had poor efficacy. In these patients, UDCA was stopped just before the ezetimibe treatment. The degree of liver injury was assessed by pretreatment measurement of alanine aminotransferase (ALT), which was elevated over 50 U/L in all patients. All patients received 10 mg of oral ezetimibe daily for more than 12 months although serum cholesterol levels were not morbidly high. None of the patients underwent subsequent therapy during the follow-up period. Six months after starting ezetimibe, serum levels of ALT was significantly decreased from baseline (70.125 ± 24.608 vs. 36.635 ± 19.405, p = 0.0135) and the levels were kept constant for 12 months (70.125 ± 24.608 vs. 34.250 ± 12.612, p = 0.0021) (Table 1, Figure 1B). In 4 patients, ALT levels dropped to within the normal range (<30 U/L) both 6 and 12 months after the start of therapy (data not shown). Decline rates of ALT levels at 6 and 12 months were -45.25 ± 24.19% and -49.33 ± 16.09%, respectively (Figure 2). Naturally, ezetimibe treatment decreased serum levels of LDL-cholesterol by 17.10 ± 11.36% (at 6 months) and 18.28 ± 15.86% (at 12 months) (Figure 2), but HDL-cholesterol, triglyceride and HOMA-IR levels were unchanged (Table 1). There was no significant correlation between ALT and LDL-cholesterol. Serum γ-glutamyl transpeptidase (GGT) levels also decreased (Table 1), but the difference was not significant. Clinical course of a representative case (48-year-old male) is presented in Figure 3. Although quantification of hepatic fat content could not be performed by liver biopsy or computed tomography, no significant attenuation of fat was found ultrasonographically even after 12 months of ezetimibe treatment (data not shown).

**Table 1:** Clinical characteristics of patients, before and during ezetimibe treatment

	Baseline	6 months	12 months
Body mass index (kg/m^2^)	23.03 ± 2.04	22.81 ± 2.34	22.84 ± 2.17
ALT (IU/L)	70.13 ± 24.61	36.64 ± 19.41*	34.25 ± 12.61*
GGT (IU/L)	115.00 ± 62.28	69.50 ± 28.49	68.38 ± 28.28
Total cholesterol (mg/dL)	212.00 ± 49.01	192.25 ± 30.68*	191.38 ± 28.61*
Triglyceride (mg/dL)	98.88 ± 34.22	89.88 ± 31.56	94.88 ± 30.62
HDL-cholesterol (mg/dL)	59.13 ± 11.81	58.25 ± 12.09	61.50 ± 12.51
LDL-cholesterol (mg/dL)	137.50 ± 34.59	113.13 ± 29.68*	111.00 ± 29.65*
HOMA-IR	2.31 ± 0.25	2.24 ± 0.14	2.17 ± 0.31

Data are shown as mean ± SD

ALT, alanine aminotransferase; GGT, γ-glutamyl transpeptidase; HOMA-IR, homeostasis model assessment-insulin resistance

*p < 0.05 compared with baseline

## Discussion

The concept that the onset and development of NAFLD are associated with obesity and over-intake of calories, which are linked to oxidative stress and insulin resistance, is commonly accepted. Therefore, investigation of therapeutic interventions has largely focused on agents that modify oxidative stress and insulin sensitivity. In contrast, we recently investigated the contribution of nutritional factors in non-obese individuals with NAFLD and reported that superabundant dietary cholesterol and decreased dietary PUFAs intake might contribute to NAFLD development [5,6]. Not surprisingly, cholesterol supply and fatty acid synthesis are associated on a stream of liver × receptor α (LXRα)-sterol regulatory element binding protein-1c (SREBP-1c) pathway. In hepatocytes, LXRα regulates cholesterol and fatty acid metabolism, and its endogenous agonistic ligands are oxysterols [7-10]. We have previously examined expression of lipid metabolism-associated genes in NAFLD liver [11-18]. As a result, despite cholesterol overload in hepatocytes, cholesterol synthesis is activated in the NAFLD liver, meaning cholesterol metabolism is dysregulated in NAFLD. Surplus cholesterol may lead to increased levels of oxysterols, activation of the LXRα-SREBP-1c pathway and enhanced fatty acid synthesis. Furthermore, up-regulation of LXRα expression was more noticeable in non-obese rather than in obese NAFLD patients. These results might reveal new targets for the treatment of NAFLD. Of note, suppression of dietary cholesterol absorption may be a feasible option to successfully treat NAFLD, particularly in non-obese patients.

NPC1L1 located in the proximal jejunum and on the canalicular aspect of the hepatocytes is essential for the absorption/reabsorption of cholesterol and other plant sterols from the intestine and liver [19]. Animal knockout models of NPC1L1 or treatment with an inhibitor of NPC1L1-dependent cholesterol transport provides resistance against hepatic steatosis [20,21]. Accordingly, the NPC1L1 inhibitor ezetimibe, which is expected to decrease intracellular cholesterol levels and down-regulate/inactivate LXRα, can be used as novel therapeutic option for NAFLD. Based on the findings above, we tried to suppress cholesterol absorption by oral administration of ezetimibe (10 mg/day). We initiated the first pilot trial of ezetimibe with 12 months of follow-up in a group of non-obese patients with NAFLD, although their serum cholesterol levels were not morbidly high. No other medications known to alter aminotransferases or liver histology in NAFLD including statins, ursodiol or fibrates were taken by these patients during the follow-up period. Because the effects of confounding variables, especially lifestyle modifications, should be minimized, no patient had an exercise or diet programs for BMI reduction. As a result of ezetimibe treatment, all patients had a sufficient biochemical response. Serum levels of ALT and GGT as well as cholesterol were effectively lowered and the effect continued during the observation period. Especially, ALT was lowered significantly and in four of 8 patients its levels reached the normal range. However, none of these patients had evidence of ultrasonographic improvement within 12 months. Although there is a possibility that ezetimibe treatment improves insulin resistance, HOMA-IR levels were unchanged during the treatment. It may be because all patients in this study were non-obese and insulin resistance was not found at the baseline.

Would ezetimibe also be effective in obese NAFLD patients? Because diet and exercise therapies are usually prescribed simultaneously to obese patients, it is difficult to evaluate the effect of ezetimibe therapy independently. Perhaps, clinical improvement with ezetimibe alone may be insufficient because, in addition to cholesterol, other factors such as total calories, carbohydrate and visceral fat associated with adipocytokine levels and insulin resistance have strong influence on NAFLD in obese individuals. However, ezetimibe may still be a promising means for treatment.

## Conclusion

Although limited by a small number of patients, our study represents the 12 months of follow-up for non-obese patients who received ezetimibe treatment for NAFLD. Our results indicate that inhibition of cholesterol absorption has a suppressive effect on liver injury in NAFLD patients without any lifestyle modifications. We could not demonstrate an obvious reduction of hepatic fat content in ultrasonographic findings within the first year. But confirmatory evidence of improvement in images awaits further study.

## Abbreviations

NAFLD: nonalcoholic fatty liver disease; PUFA: polyunsaturated fatty acid; NPC1L1: Niemann-Pick C1 like 1; BMI: body mass index; HOMA-IR: homeostasis model assessment-insulin resistance; UDCA: ursodeoxycholic acid; ALT: alanine aminotransferase; GGT: γ-glutamyl transpeptidase; LXRα: liver × receptor α; SREBP-1c: sterol regulatory element binding protein-1c.

## Competing interests

The authors declare that they have no competing interests.

## Authors' contributions

ME and MakN participated in the experimental design, data analysis and drafting the manuscript. MoK, MaK and KK collected the clinical data. KazuhM created figures. KazuyM and ManN performed the statistical analyses.

All authors have read and approved the final manuscript.
